# Supplementary material for: Numerical study on advective fog formation and its characteristic associated with cold water upwelling
Source: PLoS One. 2022 Aug 8;17(8):e0267895. doi: 10.1371/journal.pone.0267895 (PMC9359529; doi:10.1371/journal.pone.0267895)
Supplement: S1 Fig — The rectangular with the dashed line indicates the SST adjusted area in which SST was decreased by 1 K or increased by 1 and 2 K if it is lower than 293.5 K. (PDF) [file pone.0267895.s001.pdf]

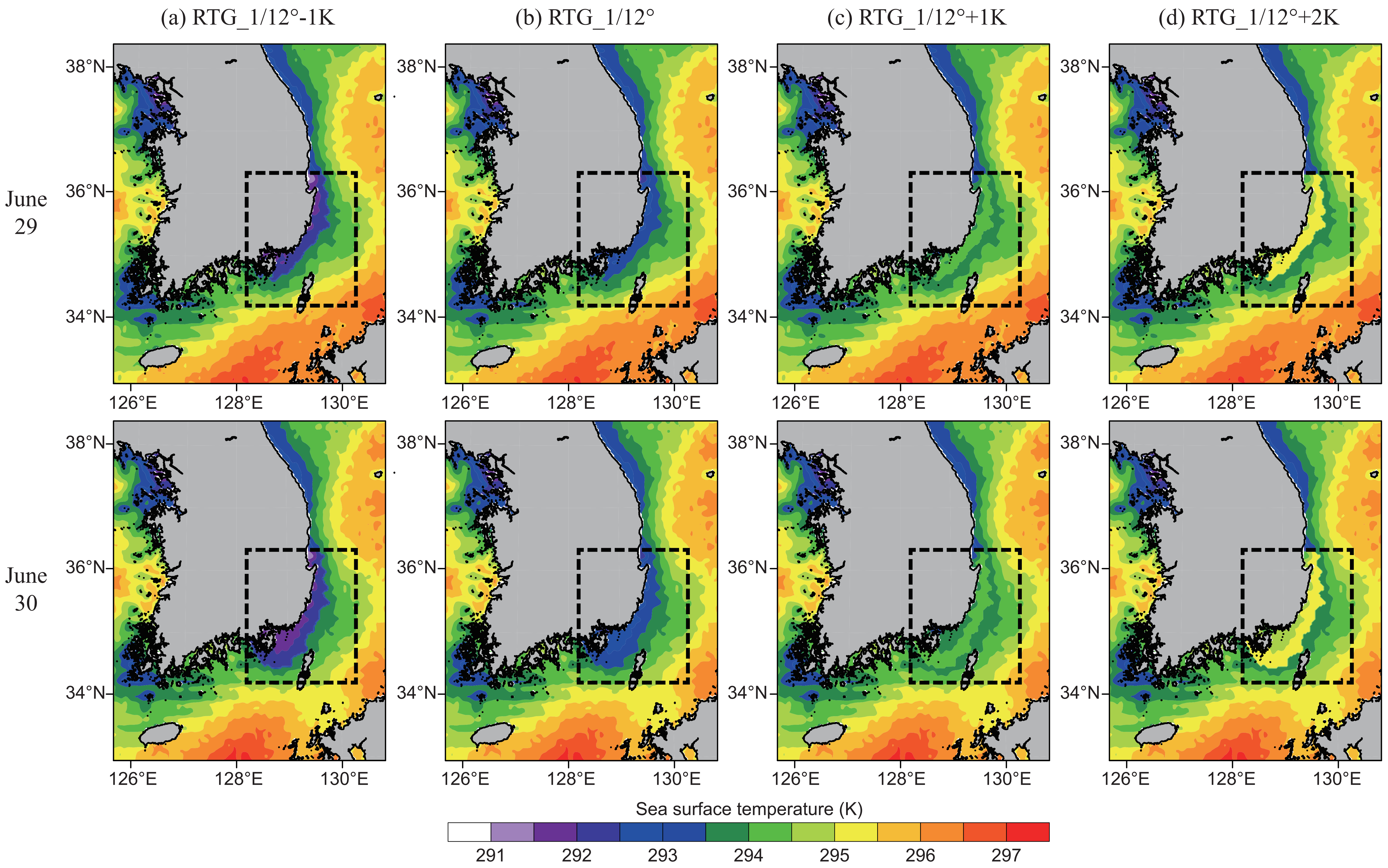

Fig S1. Horizontal distributions of sea surface temperature (SST) used for the sensitivity tests: (a) RTG_1/12°-1K, (b) RTG_1/12°, (c) RTG_1/12°+1K, and (d) RTG_1/12°+2K simulations on June 29 (top row) and June 30 (bottom row). The rectangular with dashed line indicates the SST adjusted area in which SST was decreased by 1 K or increased by 1 and 2 K if it is lower than 293.5 K.
