# Supplementary material for: Numerical study on advective fog formation and its characteristic associated with cold water upwelling
Source: PLoS One. 2022 Aug 8;17(8):e0267895. doi: 10.1371/journal.pone.0267895 (PMC9359529; doi:10.1371/journal.pone.0267895)
Supplement: S2 Fig — The rectangular with the dashed line indicates the SST adjusted area the same as in S1 Fig. (PDF) [file pone.0267895.s002.pdf]

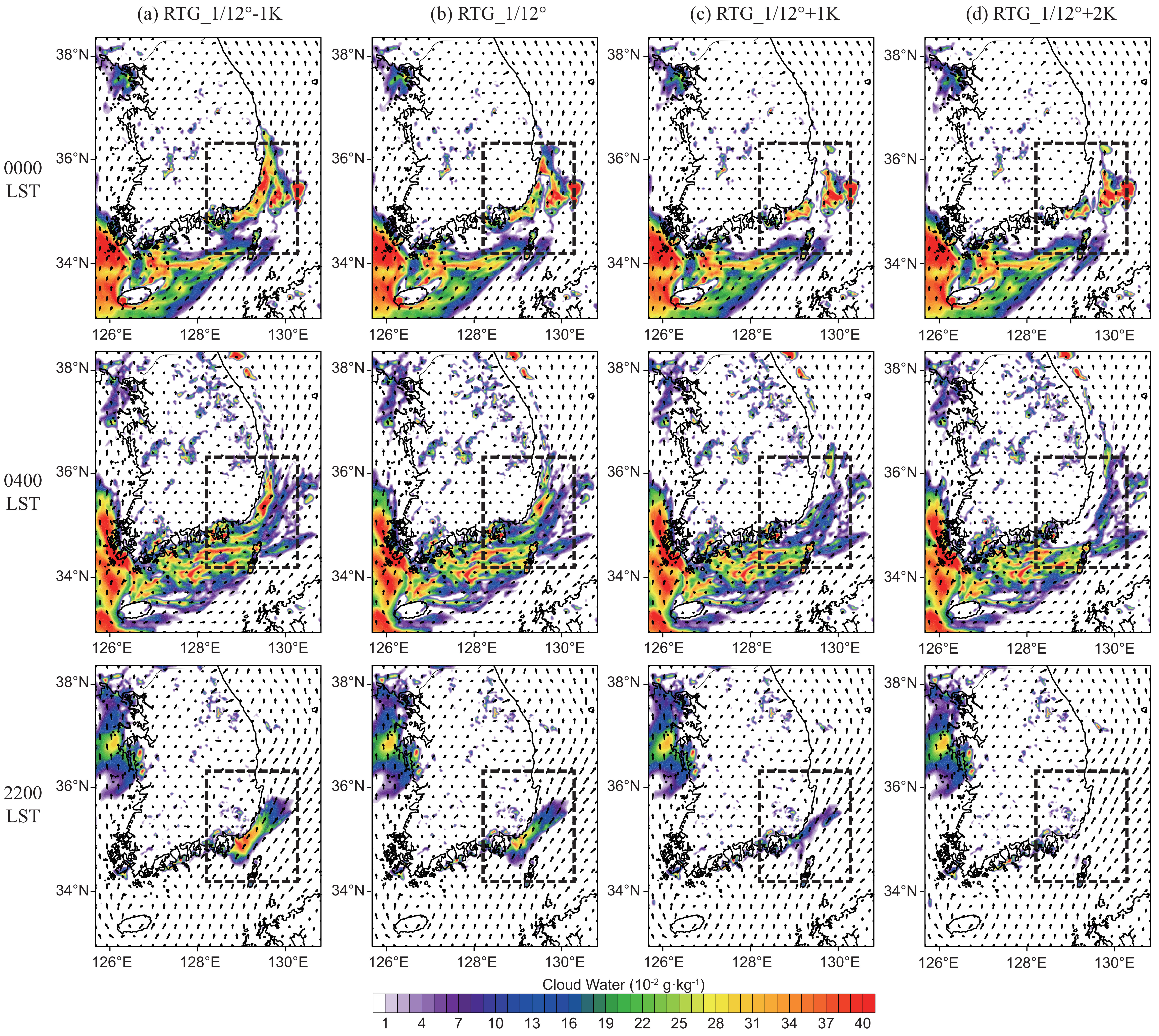

Fig S2. Spatial distributions of cloud water in the 1st model layer for the sensitivity tests: (a) RTG_1/12°-1K, (b) RTG_1/12°, (c) RTG_1/12°+1K, and (d) RTG_1/12°+2K simulations at 0000 LST (top row), 0400 LST (middle row) and 2200 LST (bottom row) on June 30, 2016. The rectangular with dashed line indicates the SST adjusted area same as in Fig S1.
