# Supplementary figures and images for: Numerical study on advective fog formation and its characteristic associated with cold water upwelling
Source: PLoS One. 2022 Aug 8;17(8):e0267895. doi: 10.1371/journal.pone.0267895 (PMC9359529; doi:10.1371/journal.pone.0267895)

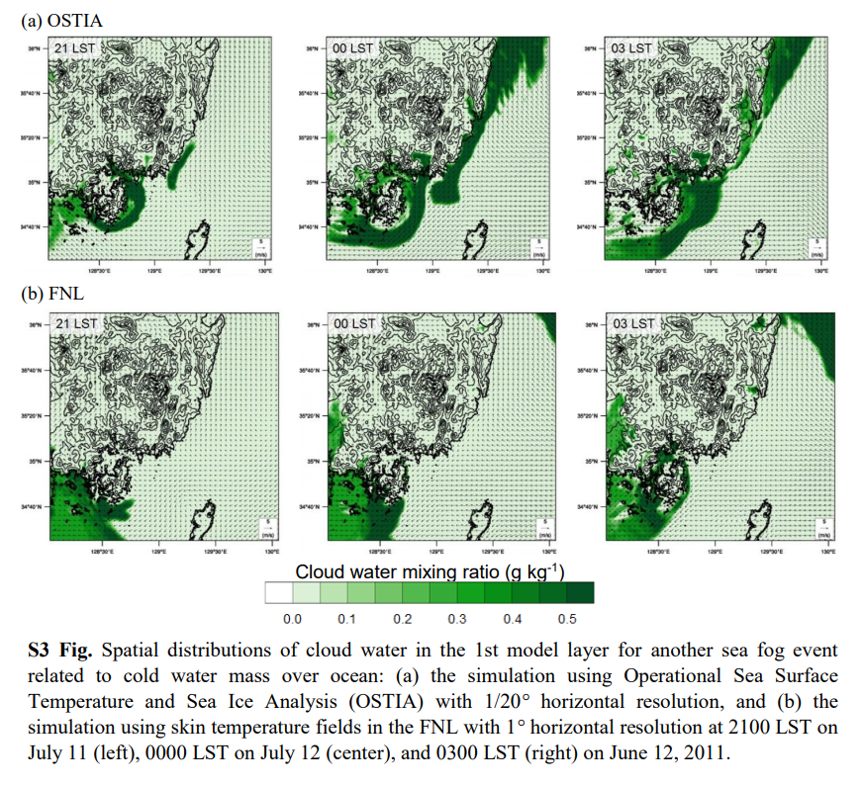

Supplement: S3 Fig — (PNG) [file pone.0267895.s003.png]
