# Supplementary material for: Numerical study on advective fog formation and its characteristic associated with cold water upwelling
Source: PLoS One. 2022 Aug 8;17(8):e0267895. doi: 10.1371/journal.pone.0267895 (PMC9359529; doi:10.1371/journal.pone.0267895)
Supplement: Attachment — Submitted filename: GI_review_A.docx [file pone.0267895.s004.docx]

Numerical study on advective fog formation and its characteristic associated with cold water upwelling

By Park et all

Submitted to PLOS ONE

Decision: Major changes

General:

Overall it is interesting paper to be followed up. Should be published but after major changes as suggested below. Intro is poorly written and missing earlier work by others. Observations are not described properly and should be given earlier even if they are limited e.g., ASOS, satellite etc. No discussion section is provided and no one knows what are the issues. Concs are limited and described properly, these concs should be itemized and explained briefly, few lines. Also, text flow because of missing/misplaced content is not performed properly, and this needs to be improved.

Major/minor issues

Ln24-27, do you have obs, why only WRF used?

Ln48; Gultepe et al AMS bulletin see for this impact

Ln49-50; that is not correct, if not validated

LN50; advection cooling, not good term

Ln52; same issue adv cooling?

Ln62; Gultepe et al 2021, lately studied marine fog from CFOG project suggested various important processes play an important role for marine fog formation.

Introduction is very limited, in fact many work done in this respect, see Gultepe et al review papers (see below), nothing mentioned.

Ln64; all know this and LN 66 most severe of what?

Ln83; what are the specific conditions are looked for? SST important all know but what is your contribution.

LN121-130; where are the observations, and how they are used?

Section 3.2 Observations; should be given first, not after model story.

Section 3.4 Comparison of simulated sea fog?? With what? Should say “verification of simulations”NEED

New Section is needed: DISCUSSIONS; I DON’T SEE.

Conclusions

Ln 326; how much properly simulated, observations were not clearly presented.

You need to show first obs and then make conclusions, if no observations then you should say it in the start of paper. But later you say ASOS observations and satellite data.

Manuscript needs in serious improvements, please also see review papers on marine fog that needs to be mentioned:

Suggested papers:

Yang, D., H. Ritchie, S. Desjardins, G. Pearson, A. MacAfee, and I. Gultepe, 2009: High Resolution GEM-LAM application in marine fog prediction: Evaluation and diagnosis. ***Weather and Forecasting***. V. 25, 727-748.

Gultepe, I., Milbrandt, J. A., and Zhou, B., 2017: Marine fog: A review on microphysics and visibility prediction. A chapter In the book of Marine Fog: Challenges and Advancements in Observations, Modeling, and Forecasting, Springer Pub. Comp. NY 10012 USA. Edited by Darko Koracin and Clive Dorman. 345-394 pp.

Gultepe, I., A.J. Heymsfield, H.J.S Fernando, E. Pardyjak, C. E. Dorman, Q. Wang, E. Creegan, S. W. Hoch,, D. D. Flagg, R.Yamaguchi, R. Krishnamurthy, S. Gaberšek, W. Perrie, A. Perelet, D.K. Singh, R. Chang, B. Nagare, S. Wagh, and S. Wang, 2021: A review of Coastal Fog Microphysics during C-FOG. Bound. Layer. Meteor.

Gultepe, I., G. Pearson J. A. Milbrandt, B. Hansen, S. Platnick, P. Taylor, M. Gordon, J. P. Oakley, and S.G. Cober, 2009: The fog remote sensing and modeling (FRAM) field project. ***Bull. Of Amer. Meteor. Soc*.**, v.90, 341-359.
