# Supplementary material for: Numerical study on advective fog formation and its characteristic associated with cold water upwelling
Source: PLoS One. 2022 Aug 8;17(8):e0267895. doi: 10.1371/journal.pone.0267895 (PMC9359529; doi:10.1371/journal.pone.0267895)
Supplement: Attachment — Submitted filename: Response_to_referee_2.docx [file pone.0267895.s005.docx]

Response to Reviewer 2

- All the line numbers in our responses are based on the revised manuscript with tracked changes.
- Summary:

#1. Because most sea fogs belong to advective fog, the difference between AT and SST is a vital factor for sea fog formation. Warm-moist air mass advection and cool sea surface help to make this difference. High-resolution SST can capture more details of coastal sea fog than low-resolution SST, it's not surprise and not a new finding. Therefore, the novelty of this article is not enough.
    - We have addressed all the suggestions and have revised the manuscript extensively according to the reviewer’s comments below. Please consider our responses and the modified manuscript. We have also analyzed the differences between air temperature (AT) and SST at the selected 7 locations in Fig 1. Instead of air temperature (AT), we used dew point temperature (Td) following Cho et al. (2000) [13], in which they examined the relationship between Td-SST and a sea fog episode using 10 years of observation data. We have added a table for this analysis and have discussed the results in the manuscript (Table 4 and line 425-437)

- Major comments:

#2. Observed facts
    a) the phenomenon of sea fog occurrence due to upwelling cold water: Please make a statistics of the spatio-temporal distribution of sea fog along the Korean coast. It's a neccessary evidence.
    - We have added a new reference Kim et al. (2019) [22] in the Introduction concerning the climatological analysis of upwelling cold water in our study area, although this was written in Korean with English abstract. (line 87-91). Also, we have revised the sentences referring Cho et al. (2000) [13] to address the statistical reports about sea fog around Korean Peninsula. (line 64-71)

b) the observed sea fog in the case study: Please show the observed sea fog (modis satellite pciture, radio soudings/CLIPSO for juding the depth of sea fog ), which is used to verify/evaluate the simulated sea fog. Visit https://worldview.earthdata.nasa.gov for modis pictures.

- Unfortunately, fog information in the study area is limited, such as the visibility over the ocean. Only the geostationary satellite, COMS 1, is available for this fog event. We have moved the supplementary figure to the manuscript to describe the observed evidence and verification of the simulated sea fog. (Fig 4, line 112-116, line 234-239, and line 353-355)

#3. Sensitivity experiments
    Table 3 shows the differeces bwtween the observed SST and the reanalysis SSTs. Even for the high-resolution RTG SST, the dieffrence can reach 2K difference and even more. So, suggest to conduct SST sensitivity experments.
    - We totally agree with the reviewer’s suggestion. We have additionally conducted sensitivity tests by increasing and decreasing the SST where the cold water masses (CWM) were shown along the southeastern coast. We found that RTG_1/12° was unable to predict any sea fog areas under conditions of warming of 2 K in a CWM area. We have discussed this results in the ‘Discussion and conclusion’ section with the supplementary figures. (S1 and S2 Figs, and line 472-482)
#4. Veritical resolution of the WRF simulation: Details of eta-levels ii not seen in the article. For sea fog simulation, vertical resolution near sea surface is very vital.
    - We have revised the paragraph in which the model configurations are explained in section ‘Numerical models’. To describe the importance of the first eta-level, we have referred Yang et al. (2019) [5], and the detail eta-level configurations are shown in Table1. (Table 1, and line 129-135)
#5. More cases: One case study is not enough.
    - We agree that one case study is not enough. Actually, we simulated a sea fog event in 2011 using the Operational Sea Surface Temperature and Sea Ice Analysis (OSTIA) SST as a high resolution dataset, and compared the predictability of sea fog with that for 1 degree FNL data. The main conclusion of this simulation regarding the impact of cold water upwelling on sea fog predictability was quite similar to that in this study. Since 8 July 2015, FNL data with a 0.25 degree resolution have been released. We therefore configured the new simulations for more recent sea fog even with high resolution meteorological input being used for the control simulation. To generalize the importance of cold water upwelling SST in the study area, we have added the results from the previous simulations in the supplementary document, and have discussed them in the section ‘Discussion and conclusions’. (S3 Fig, and line 483-489)

- Summary:

#6. The number and title is not seen below the figure in PDF manuscript. It's very inconvenient for reading.
    - We’re sorry for the inconvenience. We have added all the titles and explanations below the figure PDF.
#7. In the last figure, "cloud water mixign ratio" and "water vapor mixing ratio" are upside down.
    - We have changed this. (Fig. 9)
